# Diagnostic trajectories in child and adolescent mental health services: exploring the prevalence and patterns of diagnostic adjustments in an electronic mental health case register

**DOI:** 10.1007/s00787-019-01428-z

**Published:** 2019-11-02

**Authors:** Cliodhna O’Connor, Johnny Downs, Hitesh Shetty, Fiona McNicholas

**Affiliations:** 1 School of Psychology, University College Dublin, Dublin, Ireland; 2 NIHR Biomedical Research Centre, South London and Maudsley NHS Foundation Trust, London, UK; 3 Department of Child and Adolescent Psychiatry, Institute of Psychiatry, Psychology and Neuroscience, Kings College London, London, UK; 4 School of Medicine and Medical Science, University College Dublin, Dublin, Ireland; 5 St John of God Hospitaller Services, Dublin, Ireland; 6 Our Lady’s Hospital for Sick Children, Crumlin, Ireland

**Keywords:** Diagnosis, Child and adolescent psychiatry, Case register, Longitudinal

## Abstract

Community-based epidemiological studies show transitions between psychiatric disorders are common during child development. However, little research has explored the prevalence or patterns of the diagnostic adjustments that occur in child and adolescent mental health services (CAMHS). Understanding diagnostic trajectories is necessary to inform theory development in developmental psychopathology and clinical judgements regarding risk and prognosis. In this study, data from CAMHS clinical records were extracted from a British mental health case register (*N* = 12,543). Analysis calculated the proportion of children whose clinical records showed a longitudinal diagnostic adjustment (i.e. addition of a subsequent diagnosis of a different diagnostic class, at > 30 days’ distance from their first diagnosis). Regression analyses investigated typical diagnostic sequences and their relationships with socio-demographic variables, service use and standardised measures of mental health. Analysis found that 19.3% of CAMHS attendees had undergone a longitudinal diagnostic adjustment. Ethnicity, diagnostic class and symptom profiles significantly influenced the likelihood of a diagnostic adjustment. Affective and anxiety/stress-related disorders longitudinally predicted each other, as did hyperkinetic and conduct disorders, and hyperkinetic and pervasive developmental disorders. Results suggest that approximately one in five young service users have their original psychiatric diagnosis revised or supplemented during their time in CAMHS. By revealing the most common diagnostic sequences, this study enables policy makers to anticipate future service needs and clinicians to make informed projections about their patients’ likely trajectories. Further research is required to understand how young people experience diagnostic adjustments and their psychological and pragmatic implications.

**Electronic supplementary material:**

The online version of this article (10.1007/s00787-019-01428-z) contains supplementary material, which is available to authorized users.

## Introduction

Epidemiological data show that most psychiatric diagnoses in childhood have high comorbidity and limited temporal stability [1-6]. This means that during a young person’s engagement with Child and Adolescent Mental Health Services (CAMHS), a diagnosis once received can transition into or be supplemented by different diagnostic classifications. Such diagnostic shifts have potentially profound implications for young people and families, given diagnostic labels’ significance for making sense of emotional and behavioural difficulties [7]. Knowledge of the frequency and directions of diagnostic adjustments in CAMHS is a precondition for supporting clinicians and service users with any challenges these clinical experiences may present. Understanding diagnostic trajectories will also assist clinicians and policy makers in anticipating likely prognoses and future service needs. However, few studies have either investigated the prevalence of diagnostic adjustments in CAMHS or established the typical patterns through which they occur. The current study explores these issues using data from a London-based mental health case register.

The inter-relations between diagnostic categories are typically considered in terms of concurrent comorbidity—i.e., children who qualify for different diagnoses at the same time point [3, 4, 8-10]. Beyond concurrent comorbidity, an issue that has received less empirical attention is the way diagnostic classifications evolve across time. The limited research investigating youth diagnostic trajectories reveals that longitudinal transitions between different diagnostic categories occur frequently and at above-chance levels. For instance, a study of British school-age children found only half of children who met criteria for a psychiatric disorder at baseline retained that diagnosis at 3-year follow-up [11]. Another study, which pooled data from three large community studies, showed “all disorders predicted multiple disorder categories later in development and all disorders were predicted by at least three disorder categories at the prior developmental period” [1]. Although most diagnostic categories are subject to diagnostic transitions [1], some diagnostic sequences appear particularly prevalent. Early depression predicts later anxiety disorder and vice versa [1, 2, 12, 13]. There is also temporal cross-over between ADHD and oppositional defiant and conduct disorders [2, 10]. Early conduct problems can prefigure later anxiety and depression [10, 12, 13]. Many of these associations recapitulate those identified in the comorbidity literature [3, 4, 8-10]. However, a longitudinal lens reveals some unique nuances, for instance, while early conduct difficulties predict later mood and anxiety disorders, the reverse does not seem true [1].

There are numerous outstanding questions in the literature on longitudinal diagnostic trajectories. Minimal research has explored whether diagnostic trajectories are related to socio-demographic or clinical variables. Ford et al. [11] found that the predictors of homotypic continuity (i.e. stability of the same diagnosis across time) differed across diagnostic class: persistence of ADHD and anxiety disorders was predicted by peer relationship problems; persistent conduct disorder was predicted by intellectual disability, poor family function, socio-economic deprivation and baseline psychopathology; while no factors significantly predicted persistence of depression. Very little research enlightens the socio-demographic or clinical variables that predict heterotypic continuity, i.e. change to (rather than persistence of) particular diagnostic categories. Costello et al. [2] observed that almost all cases of heterotypic continuity (i.e. longitudinal progression between different diagnostic categories) in their sample occurred in girls, and suggested this indicated the DSM-IV diagnostic criteria used were biased towards male phenotypes. An alternative explanation is that disorders with higher female incidence (e.g., mood and anxiety disorders) also have higher diagnostic instability. Analytic strategies that isolate the independent effects of diagnostic category and socio-demographic variables such as gender are necessary to clarify the profile of service users most likely to experience diagnostic adjustments.

A further feature of the literature on diagnostic trajectories is the predominant use of community samples. While such designs have important advantages for drawing population-level conclusions, community cohort studies may not accurately reflect the diagnostic patterns that occur in clinical practice. Most community studies apply research-defined diagnostic criteria rather than recording the diagnoses children have actually received. Agreement between clinician and research diagnoses is often poor, with clinical settings showing more conservative diagnostic practice [14, 15]. Additionally, diagnostic decisions in real clinical contexts may be influenced by patient preferences, clinician biases and pragmatic considerations [16-18], to which the structured assessment criteria in research studies are less sensitive. Understanding the diagnostic trajectories that occur in clinical settings is important for numerous reasons. First, it is the diagnoses actually recorded, rather than those for which children hypothetically qualify, that influence service planning and resource distribution. Second, if recorded diagnostic trajectories differ from those revealed by community studies, it may indicate certain biases or shortcomings in clinical practice (e.g., failure to regularly re-assess young service users), or alternatively that structured research assessments lack important information gleaned from nuanced clinical interviews. Third, it is clinical diagnoses that influence the ways children and families make sense of a young person’s difficulties. Research shows psychiatric diagnoses affect young people’s self-identity in diverse ways [7]. The revision or supplementation of a diagnosis may have important social and emotional repercussions for service users.

A small number of studies have investigated diagnostic stability in clinical child and adolescent populations [19-23]. These studies indicate that psychotic disorders typically have highest temporal stability, followed by internalising disorders, externalising disorders and personality disorders [20, 22, 23]. Overall, temporal reliability of diagnosis in clinical practice tends to be low. For instance, a study in a Canadian hospital found poor correspondence (average ϰ = 0.23) between the first and last primary diagnoses recorded in children’s clinical files: reliability was higher with shorter (0–1 year) intervals between first and last diagnoses (ϰ = 0.34), but extremely poor when the interval stretched to 4 years (ϰ = 0.08) [21]. A US study of diagnostic stability in children who experienced multiple hospitalisations over a 9-year period found similarly poor reliability across diagnostic episodes (ϰ = 0.37), despite patients typically being assigned to the same clinical team across hospitalisations [23]. These studies confirm diagnostic adjustments are common in clinical practice. However, the literature on diagnostic reliability in clinical contexts shows numerous limitations, with the few studies that exist relying on small (*n* < 100) samples [20, 22, 24], recruiting participants from a single clinical (usually inpatient) setting [20-23], and/or exclusively focusing on one diagnostic class [19, 24-26]. Finally, individual studies define and measure stability in different ways, usually in terms of prospective concordance, retrospective concordance or kappa coefficients. While these measures offer useful information [23], none facilitates easy inference of the proportion of CAMHS attendees who experience modification of their diagnosis during their service engagement. Furthermore, the extant literature on diagnostic stability in child and adolescent mental healthcare does not provide detailed information on typical diagnostic sequences, i.e. the diagnoses that tend to precede and follow each other. Acquiring this information is important for prognosis and service planning.

The current study represents the first large-scale study of diagnostic trajectories in child psychiatric clinical records, which incorporates data from diverse clinical settings and on multiple diagnostic classes. The analysis sought to answer the following questions:

1. What proportion of CAMHS service users undergo a longitudinal diagnostic adjustment?
2. What are the typical diagnostic trajectories that occur?
3. Are diagnostic trajectories related to any socio-demographic or clinical variables?
  - What predicts the occurrence of any diagnostic adjustment?
  - What predicts addition of specific diagnostic classes?

## Methods

### Study context

Data were collected using the South London and Maudsley (SLaM) Clinical Records Interactive Search (CRIS) electronic case register. SLaM serves a local population of 1.3 million people in the London boroughs of Croydon, Lambeth, Lewisham and Southwark. SLaM provides care to over 50,000 people annually in more than 230 inpatient, outpatient and community mental health services. CAMHS is one of SLaM’s largest services and a monopoly provider of community-based NHS mental health services for children within the catchment area [27, 28]; a census on a single day in 2014 identified 5765 active cases [29].

All SLaM clinicians record clinical notes in a standardised electronic system. These data are de-identified and uploaded to the CRIS database for approved research use. The CRIS research system is authorized by an independent Research Ethics Committee and the current study was approved by the CRIS oversight committee.

### Sample

The sample comprised children who received their first diagnosis in SLaM CAMHS within the data collection window of 01/01/10–31/12/17. Data extraction for each case began at their first recorded diagnosis and ended at the earliest of (a) window end date (b) date of 18th birthday or (c) date of death. All data extracted were recorded before the person turned 18 and while they resided within the SLaM catchment area.^1^

### Measures

#### Diagnosis

In the SLaM clinical records system, one primary and up to five secondary diagnoses are recorded in structured fields populated with ICD-10 diagnostic categories.^2^ Previous analyses found 93% of active patients had a primary diagnosis recorded [29].

For the current analysis, diagnoses were clustered into ten overarching diagnostic classes: affective disorders, anxiety and stress-related disorders, conduct and impulse disorders, hyperkinetic disorders, pervasive developmental disorders, eating disorders, gender identity disorders, personality disorders, schizophrenia and related disorders, disorders of social functioning. ‘Not otherwise specified’ diagnostic codes were excluded, as were medical/neurological conditions and diagnostic classes that pilot research indicated were infrequently entered into the Primary Diagnosis field in SLaM CAMHS services (excluded diagnostic classes were somatoform disorders, dissociative disorders, substance use disorders, intellectual disability and sleep disorders). The structured diagnostic fields were electronically searched with a gazetteer of ICD-10 codes and keywords corresponding to each of the ten diagnostic classes (see Online Resources Table A.1). A ‘hit’ for any diagnostic class entailed the earliest appearance of any of the relevant codes or keywords in that clinical record.

Children’s diagnoses were sequenced into consecutive ‘index diagnoses’. The first, Index Diagnosis 1, was the earliest mention of any of the above diagnostic classes in the structured diagnostic fields. In 98.5% of cases, this was the earliest entry to the Primary Diagnosis field.^3^ Index Diagnosis 2 was defined as the earliest appearance of a diagnosis that (a) was a different diagnostic class from Index Diagnosis 1 and (b) whose earliest recorded appearance was at least 30 days after the date Index Diagnosis 1 was applied. Index Diagnosis 3 was the earliest appearance of a diagnostic class different from the prior index diagnoses, that appeared at least 30 days after Index Diagnosis 2, and so on.

The 30-day interval criterion for defining index diagnoses was determined following consultation with SLaM staff, who confirmed this allowed sufficient time to ensure all paperwork relating to a single diagnostic event was uploaded (i.e., that index diagnoses would reliably pertain to separate diagnostic events). The 30-day interval criterion, together with the condition that an index diagnosis must be the earliest mention of that diagnostic class, ensured Index Diagnoses 2 onwards represented genuinely new additions to the diagnostic record, rather than reference to comorbid secondary diagnoses applied contemporaneously with the first diagnostic event. However, the structured diagnostic fields do not stipulate whether a new diagnosis replaces or supplements the prior diagnosis. A qualitative analysis of the textual notes corresponding to each clinical record is currently ongoing to explore in more detail the clinical rationale for the addition of new diagnoses. For the present study, a diagnostic adjustment was simply operationalised as the addition of a new diagnosis, which may involve either the revision or supplementation of previously recorded diagnoses.

#### Covariates

##### Socio-demographic data

Socio-demographic data included child gender, borough of residence, ethnicity, month of birth, ethnicity, and neighbourhood Index of Multiple Deprivation (IMD) score. The IMD is the official government-issued measure of relative deprivation for small areas in England, ranking every small area from 1 (most deprived) to 32,844 (least deprived).

##### Clinical data

Data on SLaM service use included dates of earliest and latest face-to-face clinical contacts, total number of face-to-face contacts, and number of inpatient days. Time receiving services was calculated as the number of days between first recorded face-to-face contact and the earliest of (a) CAMHS discharge date (b) date of death or (c) end of data collection window.

Where available, the search also extracted results from the standardised clinical assessment tools with greatest coverage in SLaM CAMHS: Children’s Global Assessment Scale (CGAS) and Strengths and Difficulties Questionnaire (SDQ). Where a clinical record contained more than one CGAS or SDQ report, the version closest in time to Index Diagnosis 1 was selected. The CGAS is a clinician-rated measure of the extent of impairment, with higher scores indicating better functioning (range = 1–100), and has good psychometric properties [30]. CGAS scores can be collapsed into three categories with scores ≤ 40 indicating abnormal, 41–70 borderline and > 70 normal [30] ranges. The SDQ measures a child’s performance across five dimensions of functioning (emotional symptoms, conduct problems, hyperactivity/inattention, peer problems and prosocial behaviour). Each subscale has a range of 10, with higher scores indicating more difficulties on all scales except prosocial behaviour. It can be completed by the child, parent or teacher and has established reliability and validity [31]. SDQ cutoffs based on published British norms for normal, borderline and abnormal ranges have been established for young people aged between 4–17 years [31].

### Analysis

Analysis was conducted using Stata 15. Basic descriptive statistics were computed to characterise the demographic and clinical profiles of the entire sample. To validly characterise the prevalence and patterns of diagnostic adjustments, a filter was applied to index a minimum follow-up period. The filter restricted analysis to cases with at least 1 year of data available (i.e., ≥ 365 days between first face-to-face contact and end of data collection window) and at least three recorded face-to-face contacts. Frequency statistics established the proportion of engaged service users who experienced diagnostic adjustments and the typical diagnostic sequences involved. A multivariate logistic regression was performed to identify the demographic and clinical variables that differentiated children who experienced a diagnostic adjustment from those with a single diagnosis. Further multivariate logistic regressions were conducted to establish what predicted the specific addition of the five most common diagnostic classes. All significance tests were two-tailed.

## Results

### Sample profile

#### Socio-demographic data

The search extracted 12,543 cases with at least one recorded diagnosis. The sample’s socio-demographic characteristics are presented in Table 1. The sample was roughly evenly split by gender (51.95% male, *n* = 6511). Children resided in the boroughs of Croydon (29.24%, *n* = 3667), Lewisham (27.66%, *n* = 3470), Southwark (22.07%, *n* = 2768) and Lambeth (21.03%, *n* = 2638). Of children with recorded ethnicity information, 50.76% (*n* = 5646) were classified as White/British. Average age at first diagnosis was 12.24 (SD = 3.86, range = 0.39–17.99). Median IMD was 30.85 (range = 3.93–60.58).

**Table 1:** Socio-demographic and clinical profile of the sample

Socio-demographic data					
	*N*	%			
Gender					
Male	6511	51.95			
Female	6023	48.05			
Borough of residence					
Croydon	3667	29.24			
Lewisham	3470	27.66			
Southwark	2768	22.07			
Lambeth	2638	21.03			
Ethnicity					
White/British	5646	50.76			
Black/Afro-Caribbean	4038	34.56			
Asian	516	4.42			
Other	1484	12.70			

	Mean	SD	Min.	Max.	
Age					
Years at Index Diagnosis 1	12.24	3.86	.39	17.99	

	Median	Min.	Max.		
Area deprivation					
IMD	30.85	3.93	60.58		

Clinical data					
	Mean	SD	Min.	Max.	
Service use					
Days in SLaM	1227.25	807.75	0	2905	
Number of face-to-face contact days	15.60	23.43	0	430	
Number of inpatient days	2.59	22.56	0	638	

	Mean	SD	% Normal	% Borderline	% Abnormal
SDQ (parent-completed)					
Emotional	5.22	2.79	29.1	11.9	59.0
Conduct	3.81	2.55	34.7	15.8	49.5
Hyperactivity	6.19	2.94	40.6	10.3	49.1
Peer problems	3.68	2.38	34.7	15.1	50.2
Prosocial	6.63	2.53	67.4	11.9	20.7
Total	19.33	6.81	21.1	13.5	65.4
Impact	5.33	3.53	10.7	5.4	83.8
SDQ (child-completed)					
Emotional	5.77	2.76	43.1	11.2	44.9
Conduct	3.44	2.21	55.8	13.8	30.4
Hyperactivity	5.62	2.53	47.6	13.7	38.7
Peer problems	3.40	2.20	54.7	27.6	17.7
Prosocial	7.00	2.12	76.7	10.6	12.7
Total	18.70	6.24	30.7	24.3	45.0
Impact	3.89	3.10	17.9	8.9	73.2
CGAS					
Total	54.67	11.46	7.2	84.5	8.3

	*N*	%			
Index Diagnosis 1					
Anxiety and stress-related disorders	3409	27.18			
Affective disorders	3318	26.45			
Pervasive developmental disorders	1892	15.08			
Conduct and impulse disorders	1491	11.89			
Hyperkinetic disorders	1484	11.83			
Eating disorders	489	3.90			
Disorders of social functioning	201	1.60			
Schizophrenia and related disorders	194	1.55			
Personality disorders	39	0.31			
Gender identity disorders	26	0.21			

#### Clinical data

Table 1 presents the clinical data extracted. The sample spent an average of 3.4 years (*M* = 1227.25 days, SD = 807.75) receiving SLaM services. This included an average of 15.60 (SD = 23.43) face-to-face contacts and 2.59 (SD = 22.56) inpatient days per person.

The records of 93% (*n* = 11,661) cases included a CGAS report, while 55.24% (*n* = 6929) contained a parent-completed SDQ and 33.6% (*n* = 4215) a child-completed SDQ. Table 1 presents sample means and standard deviations for all subscales, alongside the proportions scoring in clinically significant ranges.

#### Diagnoses

Table 1 presents the distribution of first diagnoses recorded (Index Diagnosis 1). The most common diagnoses were anxiety and stress-related disorders (27.18%), followed by affective (26.45%), pervasive developmental (15.08%), conduct and impulse (11.89%) and hyperkinetic (11.83%) disorders.

### Diagnostic trajectories

#### What proportion of CAMHS service users undergo a longitudinal diagnostic adjustment?

Applying the filter for a minimum follow-up period excluded 3684 cases, leaving 8859 for analysis. Of this active sample, 7154 (80.75%) did not show any longitudinal adjustment of their first recorded diagnosis. 1705 (19.25%) children recorded a second diagnosis, of a different class and at more than 30 days’ distance from their first diagnosis. 197 (2.22%) registered an additional third diagnosis and 16 (0.2%) a fourth. No case recorded more than four different diagnoses separated by 30-day intervals.

#### What are the typical diagnostic trajectories that occur?

Table 2 presents the frequency of the diagnostic classes at each index diagnostic level, and the mean age at which they were recorded. It shows that affective and anxiety and stress-related disorders are most prevalent at Index Diagnosis 1, but their representation falls at subsequent diagnostic levels. Conversely, hyperkinetic, eating, personality, and schizophrenia and related disorders account for a greater proportion of subsequent than first diagnostic assignments.

**Table 2:** Frequency of each diagnostic class at each index diagnostic level

	Index Diagnosis 1			Index Diagnosis 2			Index Diagnosis 3		
	*N*	%	Age	*N*	%	Age	*N*	%	Age
Anxiety and stress-related disorders	2353	26.56	12.54	353	20.7	14.04	33	16.75	15.08
Affective disorders	2317	26.15	13.22	346	20.29	14.72	23	11.68	14.38
Pervasive developmental disorders	1271	12.65	9.74	276	16.19	12.00	37	18.78	12.86
Conduct and impulse disorders	1139	12.86	10.76	163	9.56	12.25	16	8.12	11.38
Hyperkinetic disorders	1121	12.65	9.92	388	22.76	10.18	23	11.68	12.08
Eating disorders	336	3.79	13.53	43	2.52	15.23	12	6.09	16.62
Disorders of social functioning	159	1.79	8.84	27	1.58	11.23	5	2.54	13.95
Schizophrenia and related disorders	116	1.31	15.14	50	2.93	15.70	12	6.09	16.22
Personality disorders	27	0.3	16.51	52	3.05	16.20	34	17.26	16.22
Gender identity disorders	20	0.23	14.81	7	0.41	15.60	*n* < 5*	–	–
Total	8859	100.0	11.78	1705	100.0	12.91	197	100.0	14.27

*CRIS data security policies preclude the reporting of cell sizes where *n* < 5

A mean of 577.79 (SD = 534.25) days elapsed between Index Diagnosis 1 and Index Diagnosis 2. Index Diagnoses 2 and 3 were separated by 462.90 (SD = 458.40) days. 61.12% (*n* = 1403) of Index Diagnoses 2 were recorded by a different clinical team than recorded Index Diagnosis 1. Very few (< 1%) diagnoses were made in inpatient settings.

Table 3 shows the proportion of cases of each Index 1 diagnostic class, which subsequently acquired a diagnostic addition. The diagnostic classes most likely to be followed by a second index diagnosis were disorders of social functioning (30.19%), conduct and impulse disorders (26.08%), schizophrenia and related disorders (24.14%) and affective disorders (21.75%).

**Table 3:** Frequency of diagnostic additions by Index 1 diagnostic class

Diagnostic class	Total *N* at Index Diagnosis 1	% (*N*) with subsequent diagnostic adjustment
Disorders of social functioning	159	30.19% (48)
Conduct and impulse disorders	1139	26.08% (297)
Schizophrenia and related disorders	116	24.14% (28)
Affective disorders	2317	21.75% (504)
Eating disorders	336	17.86% (60)
Pervasive developmental disorders	1271	17.62% (224)
Anxiety and stress-related disorders	2353	16.11% (379)
Hyperkinetic disorders	1121	14.09% (158)
Gender identity disorders	20	*n* < 5*
Personality disorders	27	*n* < 5*
Total	8859	19.25% (1705)

*CRIS data security policies preclude the reporting of cell sizes where *n* < 5

To clarify the most common diagnostic sequences, cross-tabulations calculated the number of cases where each diagnostic class at Index 1 was followed by the other diagnostic classes at Index 2. Table 4 displays these figures (for parsimony, Table 4 only presents cross-tabulations for the five most common diagnostic categories, each of which had > 1000 cases at Index Diagnosis 1). Table 4 contextualises the frequency figures in terms of their proportion of total instances of the relevant diagnostic class at Index Diagnoses 1 and 2: for instance, the 199 cases where anxiety and stress-related disorder preceded affective disorder represent 8.46% of all Index Diagnosis 1 cases of anxiety and stress-related disorder and 65.89% of all Index Diagnosis 2 cases of affective disorder. Reciprocally, a similar majority (65.41%) of Index Diagnosis 2 cases of anxiety and stress-related disorder were preceded by affective disorder. Further notable findings include the observation that 9.36% of initial diagnoses of pervasive developmental disorders were succeeded by a later diagnosis of hyperkinetic disorder, while 12.2% of children first diagnosed with conduct and impulse disorder subsequently received a diagnosis of hyperkinetic disorder.

**Table 4:** Frequency of cross-diagnostic sequences

Index Diagnosis 1	Index Diagnosis 2	*N*	% of diagnostic class at Index Diagnosis 1	% of diagnostic class at Index Diagnosis 2
Anxiety and stress-related disorders	Affective disorders	199	8.46	65.89
	Pervasive developmental disorders	67	2.85	26.91
	Conduct and impulse disorders	39	1.66	26.35
	Hyperkinetic disorders	34	1.44	8.99
Affective disorders	Anxiety and stress-related disorders	208	8.98	65.41
	Pervasive developmental disorders	68	2.93	27.31
	Conduct and impulse disorders	53	2.29	35.81
	Hyperkinetic disorders	86	3.71	22.75
Pervasive developmental disorders	Anxiety and stress-related disorders	56	4.41	17.61
	Affective disorders	28	2.20	9.27
	Conduct and impulse disorders	14	1.10	9.46
	Hyperkinetic disorders	119	9.36	31.48
Conduct and impulse disorders	Anxiety and stress-related disorders	37	3.25	11.64
	Affective disorders	53	4.65	17.55
	Pervasive developmental disorders	45	3.95	18.07
	Hyperkinetic disorders	139	12.20	36.77
Hyperkinetic disorders	Anxiety and stress-related disorders	17	1.52	5.35
	Affective disorders	22	1.96	7.28
	Pervasive developmental disorders	69	6.16	27.71
	Conduct and impulse disorders	42	3.75	28.38

#### Are diagnostic trajectories related to any socio-demographic or clinical variables?

##### What predicts the occurrence of any diagnostic adjustment?

To investigate the predictors of experiencing a longitudinal diagnostic adjustment, a binary variable was created that differentiated those with multiple index diagnoses versus those with only one recorded diagnosis. A logistic regression was fitted to establish the effects of demographic variables (gender, ethnicity, age at first diagnosis, IMD deciles), service use variables (time in SLaM deciles, total number of contact days), Index 1 diagnostic class, and mental health measures (CGAS, parent-completed SDQ). Complete data were available for 4933 children, with parent-rated SDQ scales responsible for the majority of missing data. The combination of variables significantly predicted the probability of temporal diagnostic adjustment, χ^2^(21) = 935.87, *p* < 0.001. Full results are presented in Table 5.

**Table 5:** Binary logistic regression predicting likelihood of undergoing a diagnostic adjustment

	Odds ratio	SE	*p*	Confidence interval (lower)	Confidence interval (upper)
Demographics					
Gender (female *=* 0)	0.972	0.083	0.741	0.823	1.149
Ethnicity (White/British *=* 0)	0.799	0.061	0.003**	0.689	0.927
Age at Index Diagnosis 1	0.974	0.012	0.035*	0.950	0.998
IMD at Index Diagnosis 1 (deciles)	1.026	0.014	0.055	0.999	1.053
Service use					
Total time in SLaM (deciles)	1.487	0.034	0.000***	1.422	1.555
Total contact days	1.010	0.001	0.000***	1.008	1.012
Index 1 diagnosis (anxiety and stress-related disorders *=* 0)					
Affective disorders	1.596	0.169	0.000***	1.297	1.964
Pervasive developmental disorders	0.685	0.096	0.007**	0.521	0.900
Conduct and impulse disorders	1.361	0.177	0.018*	1.054	1.757
Hyperkinetic disorders	0.448	0.067	0.000***	0.335	0.599
Eating disorders	2.379	0.585	0.000***	1.470	3.851
Disorders of social functioning	2.079	0.551	0.006**	1.236	3.496
Schizophrenia and related disorders	0.384	0.164	0.025*	0.166	0.887
Personality disorders	0.186	0.152	0.039*	0.037	0.922
Gender identity disorders	2.097	1.524	0.308	0.505	8.713
Mental health					
CGAS	1.001	0.004	0.678	0.994	1.009
SDQ emotional	1.020	0.015	0.182	0.991	1.051
SDQ conduct	0.977	0.019	0.222	0.941	1.014
SDQ hyperactivity	1.047	0.018	0.009**	1.011	1.083
SDQ peer problems	1.033	0.018	0.063	0.998	1.070
SDQ prosocial	0.966	0.017	0.051	0.933	1.000
Constant	0.016	0.006	0.000***	0.007	0.033
Model χ^2^	935.87***				
Pseudo *R*^2^	0.172				
*N*	4933				

*p < 0.05

**p < 0.01

***p < 0.001

The likelihood of diagnostic adjustment was positively related to longer time in SLaM (OR = 1.49, *p* < 0.001, CI 1.42–1.56) and more contact days (OR = 1.01 *p* < 0.001, CI 1.01–1.01). Younger age at first diagnosis increased the probability of diagnostic adjustment (OR = 0.97, *p* = 0.04, CI 0.95–0.1). The only socio-demographic variable to reach significance was ethnicity, with White/British children more likely to experience a diagnostic adjustment than other ethnic groups (OR = 0.8, *p* < 0.01, CI 0.69–0.93).

The diagnostic class recorded at Index Diagnosis 1 significantly affected the likelihood of undergoing a temporal adjustment. Relative to children with a first diagnosis of anxiety and stress-related disorders, there was a lower likelihood of subsequent diagnostic adjustment if the first diagnosis was pervasive developmental (OR = 0.69, *p* < 0.01, CI 0.52–0.9), hyperkinetic (OR = 0.45, *p* < 0.001, CI 0.34–0.6), schizophrenia and related (OR = 0.38, *p* = 0.03, CI 0.17–0.89) or personality (OR = 0.19, *p* = 0.04, CI 0.04–0.92) disorders. Relative to children with an initial anxiety and stress-related disorder, diagnostic adjustments were more likely if the first diagnosis was an affective (OR = 1.6, *p* < 0.001, CI 1.3–1.96), conduct and impulse (OR = 1.36, *p* = 0.02, CI 1.05–1.78), social functioning (OR = 2.08, *p* < 0.01, CI 1.24–3.5) or eating (OR = 2.38, *p* < 0.001, CI 1.5–3.85) disorders.

At a symptomatic level, a likelihood of diagnostic adjustment was related to higher levels of hyperactivity/inattention (OR = 1.05, *p* < 0.01, CI 1.01–1.08) and marginally to poorer prosocial behaviour (OR = 0.97, *p* = 0.05, CI 0.93–1). Diagnostic adjustments were not significantly predicted by the remaining SDQ subscales, CGAS scores, gender or neighbourhood deprivation.

##### What predicts addition of specific diagnostic classes?

Further logistic regressions were performed to establish what predicts the addition of specific diagnostic classes at Index Diagnosis 2. These included the same set of socio-demographic and clinical predictor variables as the previously reported model, but diagnostic classes (in both predictor and outcome variables) were restricted to the five most common classes (affective, anxiety and stress-related, conduct and impulse, hyperkinetic and pervasive developmental disorders) to allow sufficient cases for analysis.^4^ Full results are presented in Online Resources (Tables A.2–A.6), with significant predictors summarised here.

Index 2 affective disorders were significantly predicted by female gender (OR = 0.62*, p* = 0.02, CI 0.41–0.93), older age at first diagnosis (OR = 1.15*, p* < 0.001, CI 1.07–1.23) and lower hyperactivity (OR = 0.88*, p* < 0.01, CI 0.8–0.96). Controlling for all other variables, Index 2 affective disorders were more likely if the first diagnosis was anxiety/stress-related disorders relative to first diagnoses of conduct/impulse (OR = 0.44*, p* < 0.01, CI 0.26–0.74) or pervasive developmental (OR = 0.32*, p* < 0.001, CI 0.17–0.6) disorders.

Index 2 anxiety/stress-related disorders were significantly more likely if the first diagnosis had been affective disorders than conduct/impulse disorders (OR = 0.42*, p* < 0.01, CI 0.25–0.71). They were also predicted by older age at first diagnosis (OR = 1.11*, p* < 0.01, CI 1.04–1.18), longer time in SLaM (OR = 1.13*, p* < 0.05, CI 1–1.27), more emotional symptoms (OR = 1.14*, p* < 0.01, CI 1.06–1.23) and lower hyperactivity symptoms (OR = 0.87*, p* < 0.01, CI 0.8–0.94).

Index 2 conduct/impulse disorders were more likely with a first diagnosis of hyperkinetic disorders (OR = 2.6*, p* < 0.01, CI 1.4–4.82) relative to affective disorders. They were not related to any socio-demographic factor but were predicted by better CGAS scores (OR = 1.03*, p* = 0.01, CI 1.01–1.05), higher conduct problems (OR = 1.3*, p* < 0.001, CI = 1.15–1.46) and lower peer problems (OR = 0.87*, p* = 0.02, CI 0.78–0.97).

Index 2 hyperkinetic disorders were more likely after first diagnoses of conduct/impulse (OR = 2.18*, p* < 0.01, CI 1.36–3.48) and pervasive developmental (OR = 2.09*, p* = 0.01, CI 1.19–3.66) disorders, relative to affective disorders. They were also predicted by lower age at first diagnosis (OR = 0.9*, p* < 0.01, CI 0.84–0.96), male gender (OR = 1.97*, p* < 0.01, CI 1.3–3), fewer clinical contact days (OR = 1*, p* < 0.05, CI 0.99–1), poorer CGAS scores (OR = 0.97*, p* = 0.01, CI 0.95–0.99), lower emotional symptoms (OR = 0.81, *p* < 0.001, CI 0.75–0.88) and higher hyperactivity symptoms (OR = 1.5*, p* < 0.001, CI 1.36–1.66).

Index 2 pervasive developmental disorders were significantly more likely with a first diagnosis of hyperkinetic disorders (OR = 4.2*, p* < 0.001, CI 2.36–7.48) relative to affective disorders. They were more likely in males (OR = 1.59*, p* = 0.03, CI 1.04–2.41) and children younger at first diagnosis (OR = 0.89*, p* < 0.01, CI 0.83–0.95). They were significantly predicted by higher emotional (OR = 1.1*, p* = 0.02, CI 1.02–1.18) and peer (OR = 1.13*, p* < 0.01, CI 1.04–1.23) problems and lower conduct (OR = 0.9*, p* = 0.04, CI 0.82–0.99), hyperactivity (OR = 0.91*, p* = 0.04, CI 0.84–1) and prosocial (OR = 0.89*, p* = 0.01, CI 0.82–0.97) scores.

## Discussion

This analysis established that 19.3% of children attending London-based mental health services underwent a diagnostic adjustment, i.e. received a subsequent additional diagnosis, different from and at least 30 days after their first recorded diagnosis. This is likely an underestimate of the true prevalence of diagnostic adjustments, as the structured diagnostic fields used for this analysis may not capture all diagnostic activity pertaining to a child (e.g., diagnoses registered outside of SLaM or in CRIS free-text notes). Nevertheless, the study facilitates the first conservative estimate of the prevalence of diagnostic adjustments in CAMHS: approximately one in five young service users had their diagnoses adjusted during their engagement with services.

The study suggests the overall likelihood of experiencing a diagnostic adjustment is unrelated to gender, area deprivation or age of first diagnosis. The absence of an independent effect of gender conflicts with previous observations that most diagnostic discontinuity occurs in girls [2, 11]. The current analysis suggests such gender differences may not be due to gender per se, but because disorders with higher incidence among females (mood and anxiety disorders) account for most cases of diagnostic adjustment. The analysis indicates children identified as White/British are more likely to experience a diagnostic adjustment. This cannot be attributed to higher engagement with mental health services, since the ethnicity effect persisted independent of measures of service engagement. It is possible that Black or Minority Ethnic (BME) families interact with services in distinct ways that render diagnostic modification less likely (e.g., language difficulties or reluctance to query diagnosis). Alternatively, implicit racial/ethnic bias among clinical staff [32] could affect their interactions with BME families and likelihood of revisiting diagnostic formulations. Further research is required to enlighten the pathways by which ethnicity affects diagnostic practice. Similar to Ford et al. [11], particular patterns of diagnostic sequencing had unique predictors, with clinical variables generally more predictive than socio-demographic characteristics.

The diagnostic trajectories revealed in this clinical records study are largely consistent with previous epidemiological research. The study validates Copeland et al. [1] finding that all disorder categories predicted different disorders later in development and vice versa. The analysis also replicated previous findings of common diagnostic sequences. The study confirms affective and anxiety disorders longitudinally predict each other [1, 2, 12, 13], as do hyperkinetic and conduct disorders [2, 10]. Consistent with previous findings of concurrent comorbidity of ADHD and autism [9, 33], the analysis established this relationship also manifests longitudinally. Importantly, the current analysis confirms these cross-diagnostic sequences persist (1) in a clinical setting, and (2) when socio-demographic and clinical variables are controlled.

The analysis indicates minimal longitudinal overlap between (a) pervasive developmental and anxiety/stress-related, affective, or conduct/impulse disorders; (b) hyperkinetic and affective or anxiety/stress-related disorders; and (c) conduct/impulse and affective or anxiety/stress-related disorders. The latter finding coheres with Copeland et al. [1] but contradicts indications from other studies that early conduct problems predict later anxiety and depression [10, 12, 13]. The divergence from previous epidemiological studies may suggest children with conduct diagnoses have distinct service experiences. For instance, it is possible these young people are more likely to disengage and hence not be re-assessed, or that staff direct less clinical attention to the emotional symptoms they experience. The lack of longitudinal progression between pervasive developmental and anxiety/stress-related disorders is also notable given previous evidence of high anxiety symptoms in ASD populations [9, 33]. This may reflect a tendency towards ‘diagnostic overshadowing’ in ASD, whereby anxiety symptoms are ascribed to an existing ASD diagnosis rather than distinct mental health disorders [34, 35].

Research on diagnostic stability and instability is complicated by inconsistent methodological approaches regarding the degree of diagnostic drift that represents a meaningful diagnostic discontinuity [11]. The current analysis adopted a conservative operational definition of diagnostic adjustment, which required recording of a diagnosis from a different high-level diagnostic class. Shifts between specific diagnoses within each class (for example, between bipolar and unipolar depression [36] or obsessive compulsive disorder and generalised anxiety disorder [19]) were not classified as diagnostic adjustments in this study and should be a focus of future research. On the other hand, the current study may be seen as liberal in adopting a minimum time period for diagnostic adjustment of just 30 days (although the average interval between first and second diagnosis was much longer, at almost 2 years). Future research should clarify how such methodological parameters affect estimates of stability. The field would benefit from more consensus regarding the most appropriate ways to define and measure diagnostic (in)stability in clinical data.

The analysis was subject to a number of limitations. The 30-day interval criterion meant the dataset did not include complete information on young people’s diagnostic histories, as additional diagnoses that may have been applied at less than 30 days from the original index diagnosis were excluded, as were comorbid secondary diagnoses applied contemporaneously with the index diagnosis. Further, the clinical validity of the diagnoses recorded in this dataset is unclear. However, the aim of this research was to explore patterns of actual diagnostic practice, rather than psychopathological development per se. It is recorded diagnoses, even if invalid, that influence service delivery and service user understanding. The fact the trajectories identified in this study overlap with those from previous epidemiological research provides some confidence in the overall validity of these clinical diagnoses. A case-by-case evaluation of the clinical validity of diagnostic adjustments may yield interesting findings that enlighten the reasons behind these clinical decisions.

It is important to note the paucity of data on factors pertaining to the service, rather than child, that may predict diagnostic adjustments. For instance, as data on the individual clinicians who made diagnoses were not available, it was not possible to explore whether diagnostic practice varied across professional disciplines. Notably, 61% of second diagnoses were recorded by a different clinical team than had recorded the first diagnosis. This is partly due to referral to specialist teams for assessment and diagnosis of particular disorders (e.g., ASD). However, it may also indicate diagnostic adjustments are likely when a child enters new clinical environments with different staff, cultures and agendas. Future research should expand investigation of how variables such as team culture, institutional priorities and professional discipline may contribute to variation in diagnostic practice.

A further and related limitation is that, due to the reliance on structured diagnostic fields, the clinical rationale behind diagnostic adjustments remains opaque. As previously discussed, the data available did not discriminate between diagnostic adjustments that were intended to replace or supplement prior diagnoses. There are various reasons why new diagnoses may be ascribed. New diagnoses could reflect genuine change in symptomatology, revelation of previously undetected symptoms, or correction of prior diagnostic errors. Diagnostic adjustments may also represent indeterminate or atypical cases that induce diagnostic dilemmas. They can reflect evolutions in clinical knowledge or diagnostic instruments. A new diagnosis can be applied for pragmatic reasons, such as securing access to educational resources. Finally, diagnostic adjustments could reflect individual clinicians’ different personal, cultural and professional outlooks. Qualitative work with the textual clinical notes held by CRIS is ongoing to explore the various reasons new diagnoses are added to clinical files.

Further limitations of this study are common across research with case registers. Although registers have the advantage of reflecting real clinical activity, data quality depends on clinicians’ recording practices, which are often inconsistent and incomplete [37]. The SLaM electronic records system was implemented in 2006 and is now well embedded within the service, with audits showing the structured diagnostic fields used for this analysis are well populated [29]. Further concerns relate to generalisability. Extrapolation from the data is limited by its generation by a single service provider. This notwithstanding, SLaM provides a large and diverse range of clinical services, and CRIS represents one of the largest psychiatric case registers globally [37]. While the diverse demographic profile of SLaM’s catchment area is representative of many urban populations [29], international generalisation is impeded by the unique UK healthcare structure, with most services state-provided. However, this policy context is also an empirical advantage as the relative scarcity of private services means CRIS claims near-total coverage of mental healthcare provision in its catchment area [37]. This offers confidence the data collected comprehensively document children’s pathways through services.

## Conclusions

As the first large-scale study of diagnostic trajectories in CAMHS clinical practice, this study provides novel data regarding prevalence and typical presentation of diagnostic adjustments. This will enable policy makers to anticipate future service needs and clinicians to make informed projections about their patients’ likely trajectories. Understanding the typical patterns of diagnostic trajectories is necessary to both inform theory development in developmental psychopathology, and assist clinicians making judgements regarding risk and prognosis [12]. It is also important for developing therapeutic strategies sensitive to longitudinal changes in children’s symptom profiles and diagnostic classifications, and the socio-emotional challenges these changes may present. By highlighting the frequency of diagnostic adjustments, the research may also inform ongoing debate about the reliability and validity of diagnostic classifications in child psychiatry [38]. The study’s most important implications relate to the well-being of the young service users involved. This study suggests one-fifth of children attending CAMHS experience a longitudinal diagnostic adjustment, yet no research has explored how diagnostic adjustments are communicated to young people and their families or the social, emotional or pragmatic effects they may have. A new diagnosis could facilitate greater self-understanding and access to necessary clinical and educational services; it is equally possible the revision or supplementation of an established diagnosis could cause confusion, distress or loss of trust in services. Understanding the range of effects diagnostic adjustments have for the lives of the young people affected should be a priority for future research.

## Electronic supplementary material

Below is the link to the electronic supplementary material.

Supplementary file1 (DOCX 38 kb)

## Footnotes

1 This criterion ensured access to relatively complete clinical records, as it excluded (for instance) young people who spent time in a SLaM specialist inpatient unit but who accessed community services elsewhere.

2 Entering a primary diagnosis is mandatory before any secondary diagnoses can be added. Clinicians can override previous diagnostic entries; however, the overwritten data remain accessible to researchers on the CRIS database.

3 In cases where the Primary Diagnosis field was populated by diagnoses outside the above list (e.g., diagnoses related to substance dependence or intellectual disability), the earliest mention of any of the included diagnostic classes in the Secondary Diagnosis fields represented Index Diagnosis 1. If neither the Primary nor Secondary Diagnosis contained reference to any of the ten diagnostic classes, that case was not extracted for analysis. Cases with no entries in the structured diagnostic fields were similarly excluded.

4 Note that for the regressions with each Index 2 diagnostic class as an outcome variable, cases where that diagnostic class was recorded as the Index 1 diagnosis were excluded (because the data extraction strategy precluded these cases from registering that same diagnostic class at Index 2, so including them would have biased the analysis).
