# Supplementary material for: Diagnostic trajectories in child and adolescent mental health services: exploring the prevalence and patterns of diagnostic adjustments in an electronic mental health case register
Source: Eur Child Adolesc Psychiatry. 2019 Nov 2;29(8):1111–23. doi: 10.1007/s00787-019-01428-z (PMC7369254; doi:10.1007/s00787-019-01428-z)
Supplement: Supplementary file 1 — Supplementary file1 (DOCX 38 kb) [file 787_2019_1428_MOESM1_ESM.docx]

**Online resources**

**Table A.1**: Gazzetteer used to define diagnostic classes

| **Diagnostic Class** | **ICD-10 codes** | **Keywords** |
| --- | --- | --- |
| Affective Disorders | F30; F30.0-F30.9, F31; F31.0-F31.9; F32; F32.0-F32.9; F33; F33.0-F33.9; F34; F34.0-F34.9; F38; F38.0-F38.8; F39; F93; F93.8; F93.9 | ‘bipolar'; 'depressive disorder'; 'depressive episode'; 'mild depression'; 'moderate depression'; 'severe depression'; 'profound depression'; 'reactive depression'; 'atypical depression'; 'endogenous depression'; 'recurrent depression'; 'persistent depression'; 'depression NOS'; 'depression not otherwise specified'; 'manic episode'; 'mania'; 'mood disorder'; 'affective disorder'; 'cyclothymia'; 'dysthymia'; 'cyclocthymic disorder'; 'dysthymic disorder'; 'affective disorder'; 'mood disorder'; 'emotional disorder'; 'disruptive mood dysregulation disorder'; 'MDD' |
| Anxiety & Stress-Related Disorders | F40; F40.0-F40.9; F41; F41.0-F41.9; F42; F42.0-F42.9; F43; F43.0-F43.9; F93.0; F93.1; F93.2 | ‘anxiety disorder'; 'phobia'; 'agoraphobia'; 'phobic disorder'; 'panic disorder'; 'GAD'; 'obsessive-compulsive disorder'; 'obsessive compulsive disorder'; 'OCD'; 'post-traumatic stress disorder', 'posttraumatic stress disorder'; post traumatic stress disorder'; 'PTSD'; 'stress disorder'; 'adjustment disorder' |
| Eating Disorders | F50; F50.0-F50.9; F98.2; F98.3 | ‘eating disorder'; 'anorexia'; 'bulimia'; 'ED'; 'body dysmorphic disorder'; 'Avoidant/Restrictive Food Intake Disorder'; 'ARFID', 'binge-eating disorder'; 'pica'; 'rumination disorder' |
| Personality Disorders | F60; F60.0-F60.9; F61 | ‘personality disorder'; 'PD'; 'disorder of personality' |
| Gender Identity Disorders | F64; F64.0-F64.9 | ‘gender identity disorder'; 'gender dysphoria'; 'GID' |
| Pervasive Developmental Disorders | F84; F84.0-F84.9 | ‘pervasive developmental disorder'; 'autism'; 'autistic'; 'Rett syndrome'; 'Rett's syndrome'; 'disintegrative disorder', 'Asperger'; 'Aspergers'; 'Asperger's'; 'ASD'; 'PDD' |
| Hyperkinetic Disorders | F90; F90.0-F90.9 | ‘hyperkinetic disorder'; 'hyperkinetic conduct disorder'; 'attention-deficit hyperactivity disorder'; 'attention-deficit/hyperactivity disorder'; 'attention deficit hyperactivity disorder'; 'attention deficit/hyperactivity disorder'; 'attention-deficit disorder'; 'attention deficit disorder'; 'ADHD'; 'ADD' |
| Conduct & Impulse Disorders | F91; F91.0-F91.9; F92; F92.0-F92.9; F63; F63.0-F63.9 | ‘conduct disorder'; 'oppositional defiant disorder'; 'disorder of conduct'; 'pyromania'; 'kleptomania'; 'intermittent explosive disorder'; 'impulse disorder' |
| Disorders of Social Functioning | F94; F94.0-F94.9 | ‘disorder of social functioning'; 'attachment disorder'; 'selective mutism' |
| Schizophrenia & Related Disorders | F20; F20.0-F20.9; F21; F22; F22.0-F22.9; F23; F23.0-F23.9; F24; F25; F25.0-F25.9; F28; F29 | ‘schizophrenia'; 'schizophrenic'; 'schizotypal'; 'delusional disorder'; 'psychosis'; 'psychotic'; 'schizoaffective'; 'schizophreniform'; 'thought disorder' |

**Table A.2**: Binary logistic regression predicting likelihood of an Index 2 diagnosis of Affective Disorder

|  |  | *Odds ratio* | *SE* | *p* | *Confidence Interval (Lower)* | *Confidence Interval (Upper)* |
| --- | --- | --- | --- | --- | --- | --- |
| Demographics | Gender (Female=0) | 0.615 | 0.129 | 0.021* | 0.407 | 0.928 |
|  | Ethnicity (White/British =0) | 1.057 | 0.203 | 0.773 | 0.726 | 1.539 |
|  | Age at Index Diagnosis 1 | 1.145 | 0.041 | 0.000*** | 1.068 | 1.228 |
|  | IMD at Index Diagnosis 1 (deciles) | 0.991 | 0.035 | 0.799 | 0.926 | 1.061 |
| Service use | Total time in SLaM (deciles) | 1.018 | 0.073 | 0.800 | 0.884 | 1.173 |
|  | Total contact days | 1.002 | 0.002 | 0.380 | 0.998 | 1.005 |
| Index 1 diagnosis (Anxiety & Stress-Related Disorders=0) | Conduct & Impulse Disorders | 0.442 | 0.117 | 0.002** | 0.263 | 0.744 |
|  | Hyperkinetic Disorders | 0.569 | 0.196 | 0.101 | 0.289 | 1.117 |
|  | Pervasive Developmental Disorders | 0.320 | 0.103 | 0.000*** | 0.170 | 0.603 |
| Mental health | CGAS | 1.011 | 0.009 | 0.225 | 0.993 | 1.030 |
|  | SDQ Emotional | 1.061 | 0.044 | 0.157 | 0.977 | 1.151 |
|  | SDQ Conduct | 0.958 | 0.049 | 0.408 | 0.866 | 1.060 |
|  | SDQ Hyperactivity | 0.877 | 0.039 | 0.003** | 0.803 | 0.957 |
|  | SDQ Peer problems | 0.995 | 0.045 | 0.906 | 0.910 | 1.087 |
|  | SDQ Prosocial | 0.990 | 0.044 | 0.819 | 0.907 | 1.080 |
| Constant |  | 0.185 | 0.207 | 0.131 | 0.021 | 1.651 |
| Model χ^2^ |  | 183.19*** |  |  |  |  |
| Pseudo *R*^2^ |  | .208 |  |  |  |  |
| *N* |  | 748 |  |  |  |  |

**p < .05. **p < .01. ***p < .001*

**Table A.3**: Binary logistic regression predicting likelihood of an Index 2 diagnosis of Anxiety & Stress-Related Disorder

|  |  | ***Odds ratio*** | ***SE*** | ***p*** | ***Confidence Interval (Lower)*** | ***Confidence Interval (Upper)*** |
| --- | --- | --- | --- | --- | --- | --- |
| Demographics | Gender (Female=0) | 0.854 | 0.181 | 0.458 | 0.564 | 1.294 |
|  | Ethnicity (White/British =0) | 0.784 | 0.143 | 0.182 | 0.549 | 1.121 |
|  | Age at Index Diagnosis 1 | 1.106 | 0.037 | 0.002** | 1.036 | 1.180 |
|  | IMD at Index Diagnosis 1 (deciles) | 0.998 | 0.033 | 0.956 | 0.936 | 1.064 |
| Service use | Total time in SLaM (deciles) | 1.127 | 0.068 | 0.049* | 1.000 | 1.269 |
|  | Total contact days | 0.998 | 0.001 | 0.184 | 0.995 | 1.001 |
| Index 1 diagnosis (Affective Disorders=0) | Conduct & Impulse Disorders | 0.420 | 0.111 | 0.001** | 0.250 | 0.705 |
|  | Hyperkinetic Disorders | 0.646 | 0.222 | 0.204 | 0.329 | 1.267 |
|  | Pervasive Developmental Disorders | 1.345 | 0.366 | 0.276 | 0.789 | 2.291 |
| Mental health | CGAS | 1.014 | 0.010 | 0.141 | 0.995 | 1.034 |
|  | SDQ Emotional | 1.140 | 0.043 | 0.001** | 1.059 | 1.228 |
|  | SDQ Conduct | 0.941 | 0.044 | 0.197 | 0.858 | 1.032 |
|  | SDQ Hyperactivity | 0.866 | 0.036 | 0.001** | 0.798 | 0.941 |
|  | SDQ Peer problems | 1.015 | 0.044 | 0.726 | 0.932 | 1.106 |
|  | SDQ Prosocial | 1.044 | 0.044 | 0.298 | 0.962 | 1.133 |
| Constant |  | 0.040 | 0.041 | 0.001** | 0.006 | 0.292 |
| Model χ^2^ |  | 145.32*** |  |  |  |  |
| Pseudo *R*^2^ |  | .154 |  |  |  |  |
| *N* |  | 826 |  |  |  |  |

**p < .05. **p < .01. ***p < .001*

**Table A.4**: Binary logistic regression predicting likelihood of an Index 2 diagnosis of Conduct & Impulse Disorder

|  |  | ***Odds ratio*** | ***SE*** | ***p*** | ***Confidence Interval (Lower)*** | ***Confidence Interval (Upper)*** |
| --- | --- | --- | --- | --- | --- | --- |
| Demographics | Gender (Female=0) | 1.424 | 0.389 | 0.197 | 0.833 | 2.433 |
|  | Ethnicity (White/British =0) | 1.165 | 0.270 | 0.509 | 0.740 | 1.834 |
|  | Age at Index Diagnosis 1 | 0.961 | 0.038 | 0.324 | 0.889 | 1.040 |
|  | IMD at Index Diagnosis 1 (deciles) | 1.042 | 0.044 | 0.336 | 0.958 | 1.132 |
| Service use | Total time in SLaM (deciles) | 0.941 | 0.069 | 0.407 | 0.816 | 1.086 |
|  | Total contact days | 1.001 | 0.002 | 0.446 | 0.998 | 1.005 |
| Index 1 diagnosis (Affective Disorders=0) | Anxiety & Stress-Related Disorders | 0.945 | 0.281 | 0.850 | 0.528 | 1.693 |
|  | Hyperkinetic Disorders | 2.595 | 0.819 | 0.003** | 1.397 | 4.818 |
|  | Pervasive Developmental Disorders | 0.494 | 0.227 | 0.125 | 0.201 | 1.216 |
| Mental health | CGAS | 1.030 | 0.012 | 0.011* | 1.007 | 1.054 |
|  | SDQ Emotional | 0.981 | 0.047 | 0.688 | 0.893 | 1.078 |
|  | SDQ Conduct | 1.297 | 0.079 | 0.000*** | 1.151 | 1.462 |
|  | SDQ Hyperactivity | 0.983 | 0.055 | 0.765 | 0.881 | 1.098 |
|  | SDQ Peer problems | 0.871 | 0.050 | 0.015** | 0.779 | 0.973 |
|  | SDQ Prosocial | 1.107 | 0.061 | 0.066 | 0.993 | 1.234 |
| Constant |  | 0.011 | 0.014 | 0.000*** | 0.001 | 0.133 |
| Model χ^2^ |  | 75.74*** |  |  |  |  |
| Pseudo *R*^2^ |  | .124 |  |  |  |  |
| *N* |  | 882 |  |  |  |  |

**p < .05. **p < .01. ***p < .001*

**Table A.5**: Binary logistic regression predicting likelihood of an Index 2 diagnosis of Hyperkinetic Disorder

|  |  | ***Odds ratio*** | ***SE*** | ***p*** | ***Confidence Interval (Lower)*** | ***Confidence Interval (Upper)*** |
| --- | --- | --- | --- | --- | --- | --- |
| Demographics | Gender (Female=0) | 1.974 | 0.421 | 0.001** | 1.300 | 3.000 |
|  | Ethnicity (White/British =0) | 1.033 | 0.196 | 0.862 | 0.713 | 1.498 |
|  | Age at Index Diagnosis 1 | 0.895 | 0.029 | 0.001** | 0.839 | 0.955 |
|  | IMD at Index Diagnosis 1 (deciles) | 1.016 | 0.035 | 0.633 | 0.951 | 1.086 |
| Service use | Total time in SLaM (deciles) | 1.057 | 0.067 | 0.376 | 0.935 | 1.196 |
|  | Total contact days | 0.995 | 0.003 | 0.049* | 0.989 | 1.000 |
| Index 1 diagnosis (Affective Disorders=0) | Anxiety & Stress-Related Disorders | 0.759 | 0.217 | 0.335 | 0.434 | 1.329 |
|  | Conduct & Impulse Disorders | 2.179 | 0.521 | 0.001** | 1.364 | 3.483 |
|  | Pervasive Developmental Disorders | 2.087 | 0.597 | 0.010* | 1.191 | 3.656 |
| Mental health | CGAS | 0.973 | 0.010 | 0.009** | 0.953 | 0.993 |
|  | SDQ Emotional | 0.812 | 0.032 | 0.000*** | 0.752 | 0.876 |
|  | SDQ Conduct | 1.062 | 0.049 | 0.194 | 0.970 | 1.163 |
|  | SDQ Hyperactivity | 1.503 | 0.075 | 0.000*** | 1.362 | 1.658 |
|  | SDQ Peer problems | 0.978 | 0.042 | 0.606 | 0.898 | 1.065 |
|  | SDQ Prosocial | 1.037 | 0.045 | 0.402 | 0.952 | 1.130 |
| Constant |  | 0.220 | 0.234 | 0.153 | 0.028 | 1.757 |
| Model χ^2^ |  | 447.11*** |  |  |  |  |
| Pseudo *R*^2^ |  | .374 |  |  |  |  |
| *N* |  | 980 |  |  |  |  |

**p < .05. **p < .01. ***p < .001*

**Table A.6**: Binary logistic regression predicting likelihood of an Index 2 diagnosis of Pervasive Developmental Disorder

|  |  | ***Odds ratio*** | ***SE*** | ***p*** | ***Confidence Interval (Lower)*** | ***Confidence Interval (Upper)*** |
| --- | --- | --- | --- | --- | --- | --- |
| Demographics | Gender (Female=0) | 1.586 | 0.339 | 0.031* | 1.043 | 2.411 |
|  | Ethnicity (White/British =0) | 0.949 | 0.176 | 0.776 | 0.660 | 1.364 |
|  | Age at Index Diagnosis 1 | 0.891 | 0.030 | 0.001** | 0.834 | 0.952 |
|  | IMD at Index Diagnosis 1 (deciles) | 0.971 | 0.033 | 0.389 | 0.910 | 1.038 |
| Service use | Total time in SLaM (deciles) | 0.931 | 0.057 | 0.240 | 0.826 | 1.049 |
|  | Total contact days | 0.997 | 0.002 | 0.116 | 0.992 | 1.001 |
| Index 1 diagnosis (Affective Disorders=0) | Anxiety & Stress-Related Disorders | 1.330 | 0.322 | 0.239 | 0.827 | 2.138 |
|  | Conduct & Impulse Disorders | 1.014 | 0.283 | 0.961 | 0.587 | 1.751 |
|  | Hyperkinetic Disorders | 4.203 | 1.237 | 0.000*** | 2.361 | 7.481 |
| Mental health | CGAS | 0.996 | 0.010 | 0.677 | 0.978 | 1.015 |
|  | SDQ Emotional | 1.096 | 0.042 | 0.017* | 1.017 | 1.181 |
|  | SDQ Conduct | 0.902 | 0.044 | 0.037* | 0.819 | 0.994 |
|  | SDQ Hyperactivity | 0.914 | 0.040 | 0.040* | 0.839 | 0.996 |
|  | SDQ Peer problems | 1.134 | 0.048 | 0.003** | 1.043 | 1.233 |
|  | SDQ Prosocial | 0.892 | 0.039 | 0.009** | 0.819 | 0.972 |
| Constant |  | 2.445 | 2.498 | 0.381 | 0.330 | 18.109 |
| Model χ^2^ |  | 86.8*** |  |  |  |  |
| Pseudo *R*^2^ |  | .099 |  |  |  |  |
| *N* |  | 940 |  |  |  |  |

**p < .05. **p < .01. ***p < .001*
